# Identification of micro-RNA expression profile related to recurrence in women with ESMO low-risk endometrial cancer

**DOI:** 10.1186/s12967-018-1515-6

**Published:** 2018-05-21

**Authors:** Tiphaine de Foucher, Maria Sbeih, Jenifer Uzan, Sofiane Bendifallah, Marine Lefevre, Nathalie Chabbert-Buffet, Selim Aractingi, Catherine Uzan, Issam Abd Alsalam, Rana Mitri, Romain H. Fontaine, Emile Daraï, Bassam Haddad, Céline Méhats, Marcos Ballester, Geoffroy Canlorbe, Cyril Touboul

**Affiliations:** 1 INSERM, UMR S 938, Sorbonne University, Paris, France; 2 Department of Obstetrics and Gynaecology, Tenon University Hospital, Assistance Publique des Hôpitaux de Paris, Sorbonne University, Paris, France; 3 Department of Obstetrics and Gynaecology, Creteil Intercommunal Hospital, Université Paris Est, Paris XII, Créteil, France; 4 INSERM, UMR S 707, Sorbonne University, Paris, France; 5 Department of Pathology, Tenon University Hospital, Assistance Publique des Hôpitaux de Paris, Sorbonne University, Paris, France; 6 Department of Dermatology, Cochin University Hospital, Assistance Publique des Hôpitaux de Paris, University René Descartes, Paris, France; 7 Department of Surgery and Oncological Gynaecology, Pitié-Salpétrière University Hospital, Assistance Publique des Hôpitaux de Paris, Sorbonne University, Paris, France; 8 Institut Universitaire de Cancérologie (IUC), Paris, France; 9 Department of Pathology, Creteil Intercommunal Hospital, Université Paris Est, Paris XII, Créteil, France; 10 Cochin Institute, Inserm U1016, CNRS 8104, University René Descartes, Paris, France; 11 INSERM, UMR U965, Paris Diderot University, Paris, France

**Keywords:** Low risk endometrial cancer, Recurrence, MicroRNAs, MicroRNA-184

## Abstract

**Background:**

Actual European pathological classification of early-stage endometrial cancer (EC) may show insufficient accuracy to precisely stratify recurrence risk, leading to potential over or under treatment. Micro-RNAs are post-transcriptional regulators involved in carcinogenic mechanisms, with some micro-RNA patterns of expression associated with EC characteristics and prognosis. We previously demonstrated that downregulation of micro-RNA-184 was associated with lymph node involvement in low-risk EC (LREC). The aim of this study was to evaluate whether micro-RNA signature in tumor tissues from LREC women can be correlated with the occurrence of recurrences.

**Methods:**

MicroRNA expression was assessed by chip analysis and qRT-PCR in 7 formalin-fixed paraffin-embedded (FFPE) LREC primary tumors from women whose follow up showed recurrences (R+) and in 14 FFPE LREC primary tumors from women whose follow up did not show any recurrence (R−), matched for grade and age. Various statistical analyses, including enrichment analysis and a minimum p-value approach, were performed.

**Results:**

The expression levels of micro-RNAs-184, -497-5p, and -196b-3p were significantly lower in R+ compared to R− women. Women with a micro-RNA-184 fold change < 0.083 were more likely to show recurrence (n = 6; 66%) compared to those with a micro-RNA-184 fold change > 0.083 (n = 1; 8%), p = 0.016. Women with a micro-RNA-196 fold change < 0.56 were more likely to show recurrence (n = 5; 100%) compared to those with a micro-RNA-196 fold change > 0.56 (n = 2; 13%), p = 0.001.

**Conclusions:**

These findings confirm the great interest of micro-RNA-184 as a prognostic tool to improve the management of LREC women.

## Background

Endometrial cancer (EC) is the most common gynecologic cancer in women in developed countries. The highest estimated incidences in 2012 are in the USA and Canada (19.1/100,000) and northern (12.9/100,000) and Western Europe (15.6/100,000) [1, 2]. It is also the 14th cancer in terms of mortality accounting for 76,000 deaths/year worldwide [1]. The most frequently occurring histologic subtype is endometrioid adenocarcinoma, with a good prognosis [3]. Women are diagnosed at an early stage (stage I of the International Federation of Gynecology and Obstetrics (FIGO) classification) in 75% of the cases, when the disease is still confined to the uterus.

Among various risk stratification systems, the European Society for Medical Oncology (ESMO) system, with its four risk groups classification for early stage EC, provides the highest discrimination for both recurrence free survival (RFS) and the risk of nodal metastases [4]. However, it shows insufficient accuracy to accurately stratify recurrence risk in women with early-stage EC, as their recurrence rates range from 2 to 26% depending on several epidemiological and histological factors [5]. Thus, additional tools are needed to promote a more individualized management of early-stage EC women. In this perspective, a growing number of studies are focusing on the key role of genetics and molecular biology in EC [6].

Micro-RNAs are short non-coding RNA, ~ 22 nucleotides in length, which work at post-transcriptional level as epigenetic regulators. They can inhibit the translation process, or initiate the process of mRNA degradation [7]. They are implied in numerous process including carcinogenesis [8], where they act as tumor suppressors or activators (onco-miRs). Several micro-RNA profiles have been described in EC, in particular related to histopathological characteristics and prognosis of the tumor [9, 10]. Especially, down regulation of micro-RNA-184, and -34c-5p, involved in epithelial to mesenchymal transition (EMT), have recently been linked to the lymph node involvement in women with early-stage grade 1 and 2 EC [11]. They can be easily detected in FFPE carcinogenic specimens [12], which opens new opportunities to develop accurate diagnosis tools for tumor staging. However, few data are available about micro-RNA profiles and recurrences in low-risk EC (LREC).

## Methods

The aim of this study was to evaluate whether micro-RNA profile from LREC women can be correlated with the occurrence of recurrences and used as a tool to adapt therapeutic management.

Many of the methods related to our patient cohort, micro-RNA extraction, microarray hybridization and analysis, qRT-PCR, and minimal p-value approach have been previously published [10, 11].

### Experimental design

Approval for the present study was obtained from the local Medical Ethics Committee (CPP Ile-de-France X; 2015-01-03).

**Fig. 1:**
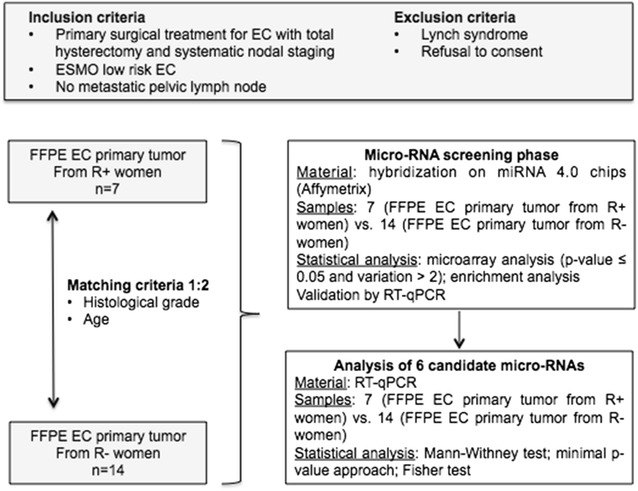
Flowchart describing the constitution of groups and the experimental design. *EC* endometrial cancer, *ESMO* European Society of Medical Oncology, *FFPE* formalin-fixed paraffin-embedded, *R+* women whose follow up showed recurrence, *R−* women whose follow up showed no recurrence, *RT* reverse transcription, *qPCR* quantitative real time polymerase chain reaction

The experimental design for profiling changes in micro-RNA according to the occurrence of recurrence in LREC FFPE primary tumor specimens is shown in Fig. 1. We included seven women, with initial LREC, who presented with histologically proven recurrences (R+) during their follow up between January 2000 and October 2014 in Créteil University Hospital and Tenon University Hospital. Their characteristics are summarized in Table 1.

**Table 1:** Histological characteristics of LREC women with recurrences

Women	Histological grade	FIGO stage	LVSI status	Nodal status	RFS (months)	Localization of recurrences
1	1	IA	Neg	15N-/15	10	Local
2	1	IA	Neg	12N-/12	34	Local
3	1	IA	Neg	7N-/7	114	Centro pelvic
4	1	IA	Neg	17N-/17	37	Local
5	1	IA	Neg	6N-/6	15	Nodal (PA) and centro pelvic
6	2	IA	Neg	26N-/26	13	Centro pelvic
7	1	IA	Neg	13N-/13	5	Distant (liver) and centro pelvic

*LREC* low risk endometrial cancer, *FIGO* International Federation of Gynecology and obstetric, *LVSI* lymphovascular space invasion, *RFS* recurrence free survival, *PA* para-aortic

According to the ESMO-ESGO-ESTRO classification, low-risk group included FIGO Stage I endometrioid carcinoma, histological grade 1–2, < 50% myometrial involvement, lympho vascular space involvement (LVSI) negative tumors. They all had disease free pelvic lymph nodes. Women underwent primary surgical treatment (including total hysterectomy, bilateral salpingo-oophorectomy, and systematic nodal staging with optional adjuvant brachytherapy according to French guidelines. Each one of them was matched according to histologic grade (grade 1 or grade 2) and age with two women without any recurrence (R−), who were used as control subjects. The exclusion criteria were as follows: Lynch syndrome (the search for a loss of expression of one of the Mismatch Repair proteins by immunohistochemistry and for tumor instability (microsatellite instability replication error repeats phenotype) were performed when EC occurred before the age 50 years or when there was a suggestive family history) and refusal of consent.

Data of women included in the study were retrospectively abstracted from prospectively maintained databases. They included age, parity, body mass index (calculated as weight in kilograms divided by the square of height in meters), comorbidities (diabetes, dyslipidemia), 2009 FIGO stage, histologic type and grade, depth of myometrial involvement, LVSI status, and time between surgery and sample analysis.

Their follow-up was conducted every 3 months for the first 2 years, every 6 months for the following 3 years, and once a year thereafter. It consisted of physical examinations, and the use of imaging techniques or histological biopsies according to the findings. Recurrences were defined according to previous reports [13]: local recurrence was defined by a vaginal vault location; central pelvic recurrence was defined by a location within the pelvis but with no involvement of the vaginal vault or of the pelvic nodes, nodal recurrence included pelvic and/or para aortic nodal locations; distant recurrence included distant metastasis (bone, liver, lung and brain). RFS was defined as the length of time from the date of primary surgery to any EC recurrence, and was censored at date of last follow-up or at date of death without recurrence.

### RNA extraction from FFPE tissues

For each woman, total RNA was extracted using the micro-RNAeasy FFPE Kit (Qiagen, Courtaboeuf, France), according to the manufacturer’s instructions.

Briefly, FFPE tissues were obtained from the hysterectomy specimens. FFPE tissue blocks were sectioned on a standard microtome (Leica-microsystems RM 2145) to generate successive 10 μm sections. A pathologist evaluated each slide, and regions of invasive carcinoma were confirmed and marked. For each sample, marked regions from several slides (number depending on the size of the invasive area, according to the manufacturer’s instructions) were microdissected using a new sterile blade, and the dissected tissues were placed immediately into an RNase-free microcentrifuge tube. After a deparaffinization step, the tissues were digested with protease and treated with DNase. After washing, the RNA, including the small micro-RNA fraction, was eluted with 20 μl distilled water. The concentrations and quality of the RNA recovered were measured using the Nanodrop 1000A spectrophotometer (Nanodrop Technologies, Wilmington, DE, USA). The median ratio of 260/280 was 1.85 (interquartile range (IQR): 1.70–1.91) and the median concentration was 143 ng/μl (IQR: 91–378).

### Microarray hybridization (GEO: GSE100078) and data analysis

Microarray analysis was conducted on 21 distinct specimens: 7 specimens from R+ women, each one of them matched with 2 specimens from R− women. Microarray hybridization on Micro-RNA 4.0 chips (Affymetrix) was conducted at the genomic platform of the Institut Cochin, Paris. After validation of the RNA quality with Bioanalyzer 2100, 1 μg of total RNA was biotin labeled following the FlashTag Biotin HSR RNA labeling kit (Affymetrix).

After overnight hybridization, the Micro-RNA 4.0 chips were washed in the Fluidic Station FS450 (Affymetrix) following a specific protocol and scanned using the GCS3000 7G. The scanned images were then analyzed with Expression Console software (Affymetrix) to obtain raw data (CEL files) and metrics for Quality Control. No apparent outliers were detected. Specific micro-RNA analysis was performed using Partek Flow software, version 3.0 Copyright, 2014 (Partek Inc., St Louis, MO, USA). CEL files were imported and normalized using robust multi-array averaging.

Micro-RNAs detected with a variation of at least 2, and with a nominal p-value ≤ 0.05, between R+ and R− women were considered to be differentially expressed, and thus selected for further analysis.

### Validated target gene and enrichment analysis

To estimate the biologic effects of the differentially expressed micro-RNAs, lists of validated target genes were determined using currently available databases, including Tarbase^®^ and Mirtarbase^®^. Gene Ontology (GO) enrichment analysis was performed on the lists using Enrichr [14, 15].

### Validation of candidate micro-RNAs with Reverse Transcription (RT) and Quantitative Real-Time PCR (qRT–PCR)

One microgram of total RNA was used in all RT reactions that were performed with the miScriptII^®^RT Kit, using the 5× miScript HiSpec Buffer method, according to the manufacturer’s instructions (Qiagen) with a Thermo Hybaid PXE 0.2 Thermal Cycler. Complementary DNA samples were stored at − 20 °C for further use.

Micro-RNA expression was analyzed by real-time PCR using miScript SYBR Green PCR Kit (Qiagen) according to the manufacturer’s instructions, with an initial activation step at 95 °C for 15 min, followed by 50 cycles of 94 °C for 15 s, 55 °C for 34 s, and 70 °C for 30 s. A final melting curve analysis was performed to verify that a single product was amplified. All steps were performed in duplicate using an Applied Biosystems^®^ 7300. The results are expressed as Ct values and normalized on the calculated median Ct of each sample (ΔCt). Micro-RNA primers were from Qiagen. Relative expression was calculated using the comparative Ct method (2^−ΔΔCt^). SNORD68, SNORD61, and RNU6 were used as endogenous controls for data normalization [10, 11].

For the results from the qRT–PCR on micro-RNA expression, data are expressed as mean ± sem. Means between two groups were compared using the Mann–Whitney test. p < 0.05 was considered to be statistically significant. GraphPad Prism version 7 was used for analysis of tissue samples (GraphPad Software, La Jolla, CA, USA).

### Optimal micro-RNA fold-change cutoffs correlated with occurrence of recurrence

For qualitative analysis, we calculated optimal cut-offs for each micro-RNA to correlate semi-quantitative expression and the occurrence of recurrence. The optimal fold-change cut-off was determined by a minimum p-value approach.

### Statistical analysis

Unless otherwise specified, data were managed with an Excel database and analyzed using R 3.1.3 software, available online.

## Results

### Clinical and pathological characteristics

The clinical and pathologic variables of the women are shown in Table 2.

**Table 2:** Epidemiologic and histologic characterizations between low risk EC women with and without recurrences

	EC FFPE primary tumor no recurrence, N = 14	EC FFPE primary tumor recurrences, N = 7	p-value
Age, median (IQR)	60 (45–82)	66 (57–76)	0.45
Diabetes n (%)	1 (7%)	1 (14%)	1
Dyslipidemia n (%)	2 (14%)	1 (14%)	1
Parity, median (IQR)	2 (0–5)	2 (1–4)	0.93
BMI, median (IQR)	28 (18–39)	26 (23–36)	0.60
Histologic grade, n (%)			1
Grade 1	12 (85%)	6 (85%)	0.70
Grade 2	2 (15%)	1 (15%)	
Tumor size (mm), median (IQR)	16 (5–50)	35 (9–45)	
Time between surgery and sample analysis (months), median (IQR)	165 (94–204)	137 (59–202)	0.52
Vaginal brachytherapy after surgery	10 (71%)	2 (28%)	0.16

*EC* endometrial cancer, *FFPE* formalin–fixed paraffin-embedded, *IQR* interquartile range, *BMI* body mass index

The median age was 66 years (interquartile range (IQR): 57–76) in the R+ group and 60 years (IQR: 45–82) in the R− group (p = 0.45). The two groups were comparable for histologic grade and tumor size. Furthermore, the comorbidities (diabetes and dyslipidemia), parity and body mass index did not differ between R+ and R− women. Neither did the treatment modalities. The median time between surgery and sample RNA extraction was 165 months (IQR: 94–204) in the R− group and 137 months (IQR: 59–202) in the R+ group (p = 0.52).

### Distinct micro-RNA signatures of LREC from R+ women

**Fig. 2:**
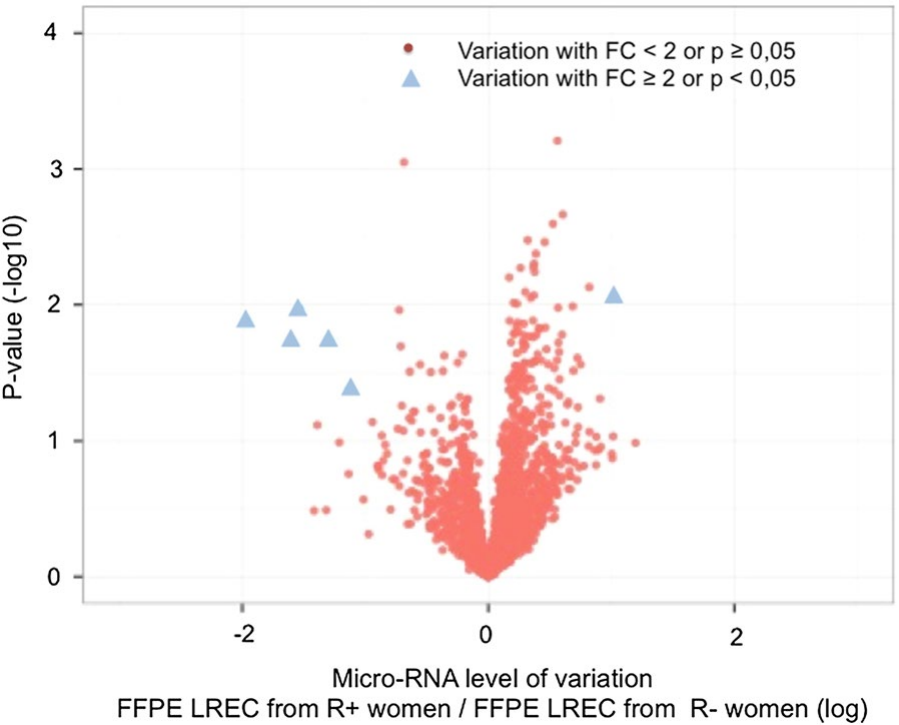
Volcano plot. Abscissa is the logarithmic value of the level of variation (LogRatio) and ordinate is the negative logarithm of the statistical value (−log (p)) of fluorescence intensities of the hybridized probes from R+ or R− samples. There was more than a 2-fold change in normalized fluorescence intensity of 6 micro-RNAs (blue triangle) between R+ vs. R− groups (p < 0.05): 5 decreased in intensity (on the left) and 1 increased in intensity (on the right). *FC* fold Change, *FFPE* formalin-fixed paraffin-embedded, *LREC* low risk endometrial cancer, *R+* women whose follow up showed recurrence, *R−* women whose follow up showed no recurrence

We focused our study on the 2578 probes containing sequences for mature micro-RNAs. As illustrated by the volcano plot representation (Fig. 2), there was more than a 2-fold change (FC) significant difference in the normalized fluorescence intensity of 6 of these micro-RNAs between the R+ and the R− groups: 5 micro-RNAs (micro-RNA-184, -497-5p, 195-5p, -7162-3p, and -196b-3p) had a decreased expression and one (micro-RNA-6080) had an increased expression in samples from the R+ group compared the R− group (Table 3).

**Table 3:** List of the down regulated and up regulated micro-RNAs

Down regulated micro-RNA			Up regulated micro-RNA		
Name	Fold change	p-value	Name	Fold change	p-value
Micro-RNA-184	− 3.91	0.013	Micro-RNA-6080	2.03	0.009
Micro-RNA-497-5p	− 3.04	0.019			
Micro-RNA-195-5p	− 2.90	0.011			
Micro-RNA-7162-3p	− 2.45	0.019			
Micro-RNA-196b-3p	− 2.18	0.041			

Fold change < -2, p-value < 0.05 and fold change > 2, p-value < 0.05, between FFPE LREC tumors from R+ patients vs. FFPE LREC tumors from R− patients

*FC* fold change, *FFPE* formalin-fixed paraffin-embedded, *LR* low risk, *EC* endometrial cancer, *R+* women whose follow up showed recurrence, *R−* women whose follow up showed no recurrence

**Fig. 3:**
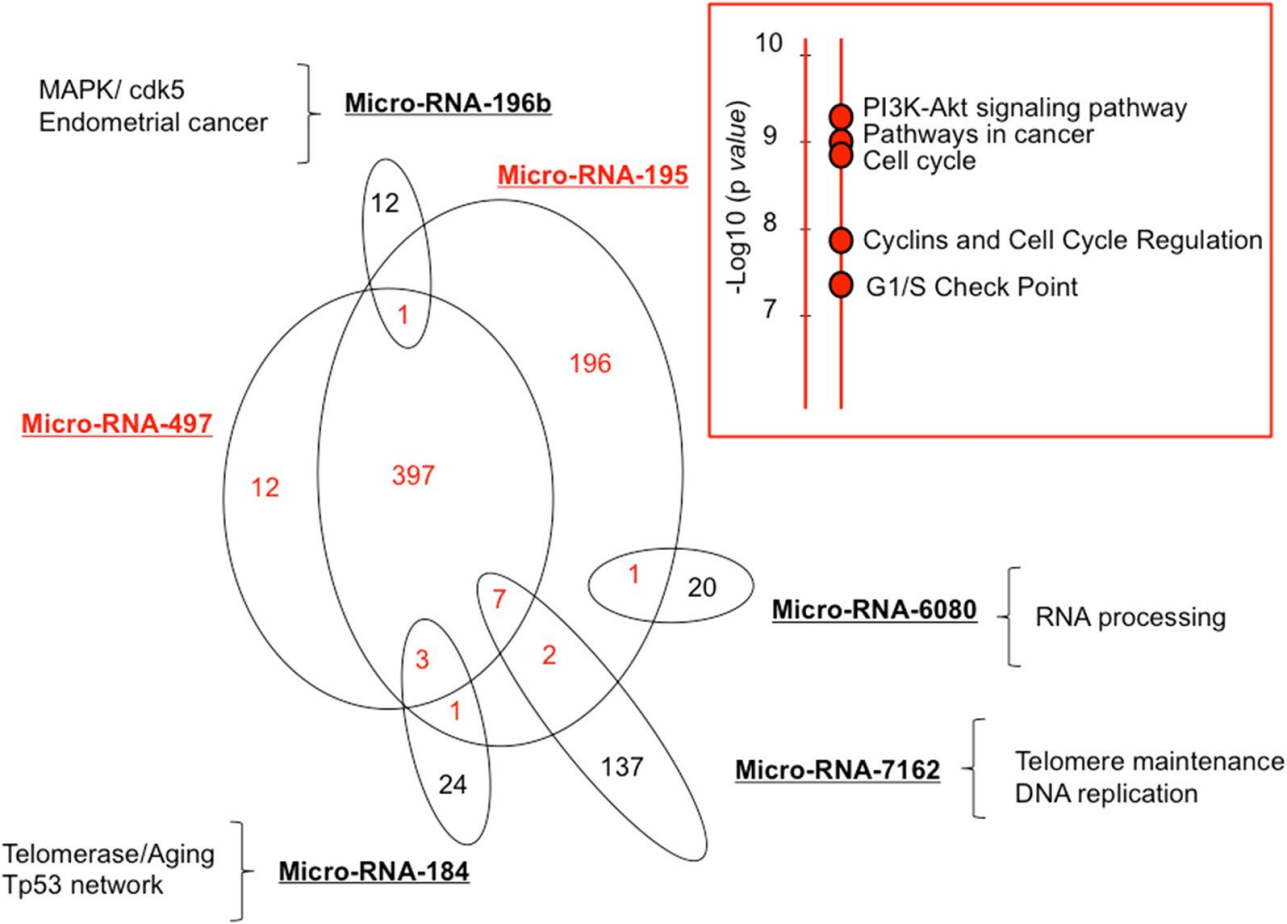
Venn diagram. It represents overlap of known validated targets for the Micro-RNAs exhibiting at least a two fold change, with a significant value (p < 0.05), in the LREC FFPE tumors from R+ patients enrichment analysis. Gene ontology enrichment analysis was performed with Enrichr. In insert, GO analysis for the common genes in red, sorted based on −log_10_(*p* value). *FFPE* formalin-fixed paraffin-embedded, *LREC* low risk endometrial cancer, *R+* women whose follow up showed recurrence, *R−* women whose follow up showed no recurrence

We extracted from the micro-RNA databases the known validated targets of the micro-RNAs exhibiting at least a 2-FC, with significant value (p < 0.05), in the LREC tumors from R+ women vs. R− women: it displays 813 genes, with the majority associated with micro-RNA-497 and micro-RNA-195. Genes involved in PI3 kinase signaling pathway, cancer and cell cycle checkpoint were specifically enriched for these two micro-RNAs. In addition, micro-RNA-7162 and micro-RNA-184 were associated with telomerase maintenance and DNA replication, micro-RNA-6080 with RNA processing, and micro-RNA-196b with endometrial cancer and MAPK/cdk5 (Fig. 3).

### Evaluation of micro-RNA expression by real–time qRT–PCR analysis

**Fig. 4:**
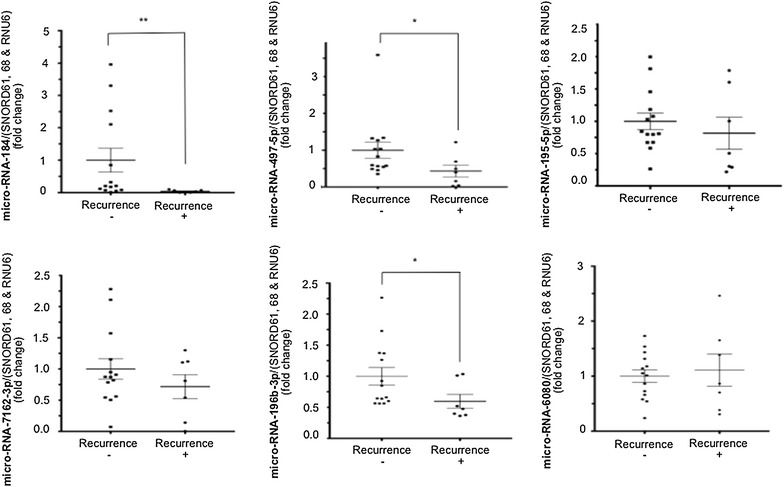
qRT-PCR assay. The expression levels of 3 microRNAs (micro-RNA-184, micro-RNA-497-5p and micro-RNA-196-3p) were significantly lower in FFPE LREC from R+ women vs. FFPE LREC from R− women. Mann–Whitney test, *p < 0.05; **p < 0.001; mean ± SEM. *FFPE* formalin-fixed paraffin-embedded, *LREC* low risk endometrial cancer, *R+* women whose follow up showed recurrence, R− women whose follow up showed no recurrence

A qRT–PCR assay was used to confirm the expres-sion of the selected micro-RNAs. The expression levels of 3 micro-RNAs (micro-RNA-184, -497-5p and -196b-3p) were significantly lower in the R+ group compared to those in the R− group. The expression levels of the 6 micro-RNAs of interest are shown in Fig. 4.

### Correlation between micro-RNA expression and recurrence in LREC

**Fig. 5:**
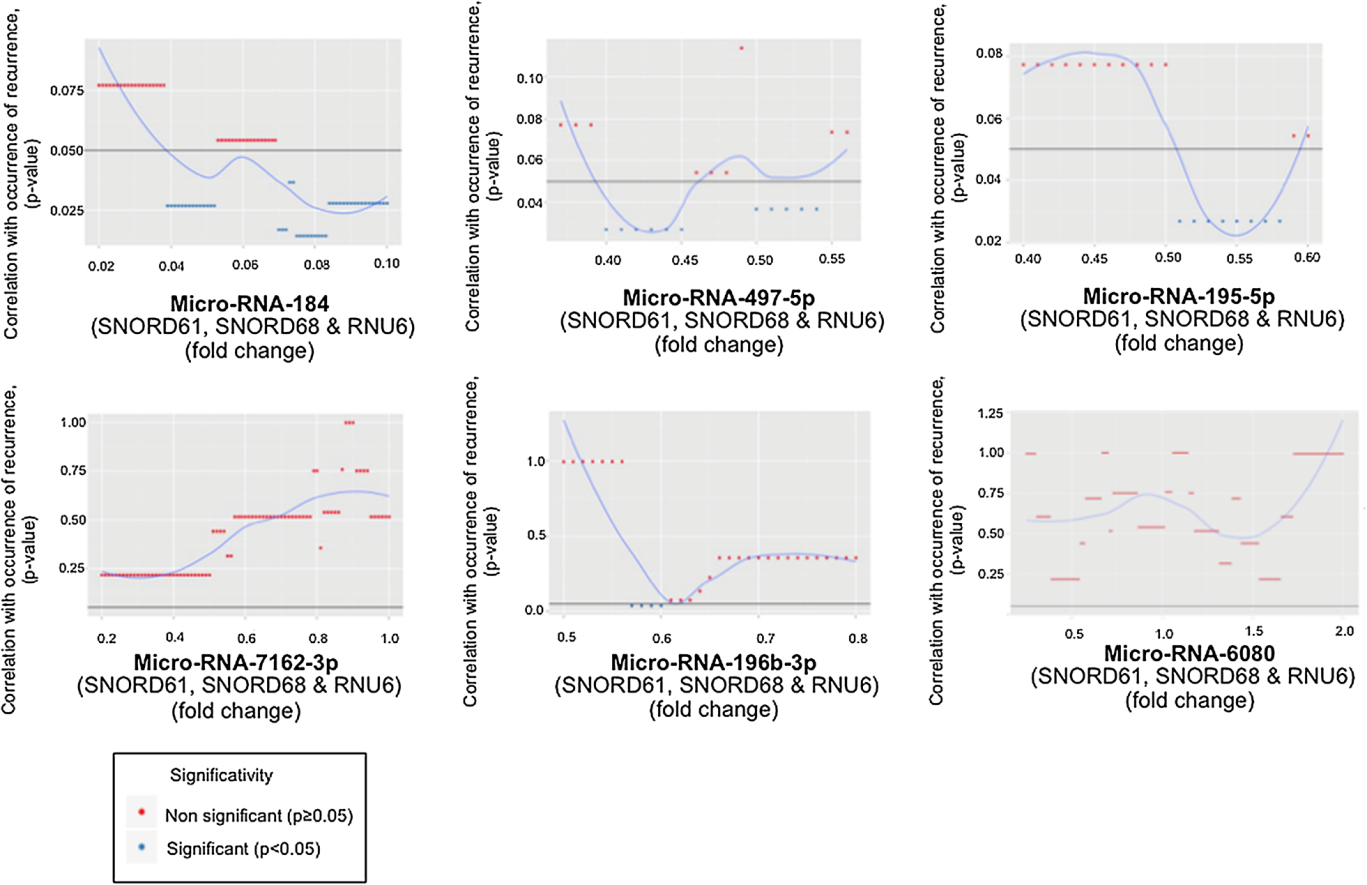
Optimal cut-offs. Optimal cut offs denoting a correlation between micro-RNA expression and the occurrence of recurrence in FFPE LREC from R+ patients vs. FFPE LREC from R− patients. *FFPE* formalin-fixed paraffin-embedded, *LREC* low risk endometrial cancer, *R+* women whose follow up showed recurrence, *R−* women whose follow up showed no recurrence

Optimal cut-offs relevant to the strongest correlation between quantitative expression of the micro-RNAs that had been selected from the previous step and the occurrence of recurrence in LREC women are summarized in Fig. 5. The FC cut-offs defined were 0.083, 0.45, 0.58 and 0.56 for micro-RNA-184, -497-5p, 195-5p, and micro-RNA -196b-3p respectively. We compared recurrence status according to the cut-offs previously determined: women with EC and a micro-RNA-184 FC < 0.083 were more likely to show recurrence (n = 6; 66%) compared to those with an micro-RNA-184 FC > 0.083 (n = 1; 8%), p = 0.016; Women with EC and a micro-RNA-497 FC < 0.45 were more likely to show recurrence (n = 4; 80%) compared with those with a micro-RNA 497 FC > 0.45 (n = 3; 19%), p = 0.025; women with a micro-RNA-196 FC < 0.56 were more likely to show recurrence (n = 5; 100%) compared to those with a micro-RNA-196 FC > 0.56 (n = 2; 13%), p = 0.001 (Table 4).

**Table 4:** Optimal fold-change cut-offs

	EC FFPE primary tumor with no recurrence, N = 14	EC FFPE primary tumor with recurrence, N = 7	p-value
Micro-RNA-184			
FC < 0.083	3 (33%)	6 (66%)	0.016
FC > 0.083	11 (92%)	1 (8%)	
Micro-RNA-497-5p			
FC < 0.45	1 (20%)	4 (80%)	0.025
FC > 0.45	13 (81%)	3 (19%)	
Micro-RNA-195-5p			
FC < 0.58	1 (20%)	4 (80%)	0.025
FC > 0.58	13 (81%)	3 (19%)	
Micro-RNA-196-3p			
FC < 0.56	0 (0%)	5 (100%)	0.001
FC > 0.56	14 (87%)	2 (13%)	

*LREC* low risk endometrial cancer, *FFPE* formalin-fixed paraffin-embedded, *FC* fold-change

## Discussion

Our results show that in LREC, R+ women have different micro-RNA profiles from R− women. Moreover, we found that recurrence status can be accurately assessed using micro-RNA expression levels on primary FFPE tumor.

The crucial issue in managing patients with early-stage EC is to determine the risk of recurrence to better adapt the therapeutic strategy. Currently, higher risk of recurrence has been described when early stage EC was associated with histological settings, including myometrial involvement > 50% [16], histological type 2 and grade 3 tumors [17, 18] and presence of LVSI [19]. However, histological factors such as histological type or LVSI status have been criticized for their lack of reproducibility [20]. Guidelines for therapies widely differ from one country to another [21–25], as they rely on various risk stratification systems (RSS) [3, 19, 21, 22, 26]. Bendifallah et al. in their comparison of five major RSS applied to a multicenter population with early-stage EC, showed that they all have a poor-to-moderate discrimination for recurrence, and are also heterogeneous in terms of classification performance [4]. Over or under estimation of these risks lead to inadequate surgical and adjuvant managements of EC women. This underlines the need for new biological markers, so as to enable a better stratification of women with early stage EC, and thus a better optimization of their treatments and follow up patterns.

Recently, Levine et al. described four molecular EC categories based on integrated genomic, transcriptomic, and proteomic analysis: p53-mutant, microsatellite instability (MSI), POLE-mutant, and no specific molecular profile [27]. Confirmation of the prognostic capacity of these four subgroups in a large randomized trial was provided by Stelloo et al. who studied 834 women from the PORTEC cohorts [28]. However, to our knowledge, there’s no study specifically focusing on biomolecular prognosis profile for LREC women. To address this issue, we assessed micro-RNA level expression in primary FFPE specimen of LREC women according to recurrence status. Microarray analysis performed after RNA extraction from 7 FFPE specimens from LREC R+ women matched with 14 FFPE specimens from LREC R− women showed a significant difference in the expression of 6 micro-RNAs: -184, -497-5p, 195-5p, -7162-3p, -196b-3p, and -6080. When studying the validated target genes of these micro-RNAs by enrichment analysis, they all appeared as exclusively involved in carcinogenic pathways, thus reinforcing our analysis. According to literature, microRNAs-184, -497-5p, 195-5p, and -196b-3p had already been described as involved in various cancers, including EC [9, 29, 30]. Furthermore, they have been described associated to the epithelial-to-mesenchymal transition (EMT), which play a key role in cell motility and metastatic spread. The recent discovery of micro-RNA-7162-3p and micro-RNA-6080 could explain why there are no currently available articles about them. In their review of the current state of science regarding microRNA functionality in EC progression, Stope et al. note that, interestingly, most micro-RNAs in EC cells have been classified as tumor suppressors, with cellular expression being suppressed by malignant processes [31]. This is consistent with our results as 5 micro-RNAs are under expressed in LREC R+ women with a FC > 2 vs. one over expressed.

After validation by RT-qPCR, we found that micro-RNA-184, -497-5p, and -196b-3p emerged as being particularly relevant to determine the occurrence of recurrence in women with LREC.

Micro-RNA-184 has been described as a tumor suppressor in various cancers (lymphoma [32], renal carcinoma [33], breast cancer [34, 35], lung cancer [36, 37], ovarian cancer [38], glioma [39], otorhinolaryngologic cancer [40–42], gastric cancer [43] or adrenocortical tumors [44]). Micro-RNA184 acts by repressing oncogenes such as TNFAIP2 [39, 43], SND1 [45], CDC25A, c-MYC and Bcl-2 [36], and by regulating the AKT/mTORC1 pathway. In EC, micro-RNA-184 was first described as under expressed by Chung et al. who profiled micro-RNA expression of 30 EC specimen FIGO stage from IA to IIIC, and 22 normal counterparts [29]. More recently, Canlorbe et al. found a strong correlation between level expression of micro-RNA-184 extracted from FFPE specimen of women with grade 1–2 EC and lymph node status [11]. In our study, all included women underwent pelvic nodal staging. They were excluded in case of positive lymph node, as it represents a major bias in the analysis of recurrences. In women with LREC, micro-RNA-184 emerges as an interesting prognostic tool, as its under expression is significantly associated with pelvic lymph node metastasis and/or recurrences.

Similarly, a decreased expression of micro-RNA-497-5p is associated to multiple myeloma [46], thyroid cancer [47], colorectal cancer [48, 49], cervical cancer [50], glioma [51], ovarian cancer [52, 53], breast cancer [54–56], bladder cancer [57, 58], hepatocellular carcinoma [59, 60], otorhinolaryngologic cancer [61, 62], pancreas cancer [63], prostate cancer [64], lung cancer [65], renal cancer [66], gastric cancer [67], melanoma [68] or astrocytoma [69]. Various studies showed that over expression of micro-RNA-497 can suppress cell proliferation and induces apoptosis through targeting paired box 3 [46], paired box 2 [52] and YAP1 [59], suppress EMT and metastasis by targeting fos related antigen-1 [48] or vascular endothelial growth factor-A [49], and reduce tumor growth and invasion by suppressing BDNF [47].

Finally, micro-RNA-196 is a known emerging biomarker for digestive tract cancers, where it plays the role of an onco-miR, as it is consistently found over expressed in digestive tract cancer tissues [70–73]. It has also been described as up regulated in otorhinolaryngologic cancer [74], glioblastoma [75], lung cancer [76], ovarian cancer [77] or osteosarcoma [78]. On the other hand, micro-RNA-196 down regulation is implicated in homeobox B7-vascular endothelial growth factor pathway, which plays an important role in cervical cancer progression [79]. It was also described as down regulated among 54 other micro-RNAs in endometrial serous carcinoma compared to normal endometrial tissue [30]. Abe et al., in their study on the global expression pattern of micro-RNA in endometriotic stroma cells, found that expression of micro-RNA-196b in endometriotic cyst stromal cells is repressed by DNA hyper methylation of the micro-RNA-196b gene, which may be involved in the development of proliferative and anti-apoptotic characteristics of endometriosis [80].

Both micro-RNA-184 and -196 have been described as tumor genesis marker. Indeed, MicroRNA 184 was also described as significantly downregulated in tissues from patients with colorectal cancer, ovarian cancer or prostate carcinoma, compared with matched normal adjacent tissues [38, 81, 82]. It was also differentially expressed in glioma tissues compared to normal brain tissues [83], and in tongue carcinomas tissues compared to paired normal tissues [84]. Micro-RNA-196 was differentially expressed in pancreatic resection specimens from carcinoma cases and its precursors vs. benign pancreatic specimens [85], in oral squamous cell carcinoma tissues (OSCC) vs. contral mucosa [86], in breast cancer samples vs. normal breast samples [87] and in colonic cancer specimens vs. para-cancerous specimens [88]. But both of them have also been described as associated with tumor recurrence, in granulosa tumors for micro-RNA-184 [89] and in OSCC for micro-RNA-196 [86]. Unfortunately, the design of our study didn’t include normal adjacent tissues nor blood samples. To confirm that these selected microRNAs are involved not only in tumor genesis but also in recurrences, further studies comparing their expression in LREC and normal sample should be completed.

In our study, we focused on post-operative FFPE EC specimen, since it is known that FFPE tumors can be used for RT-PCR-based quantitative micro-RNA profiling [9]. This enabled us to work on samples taken between 2000 and 2014, and selected among the entire specimen collections of the Pathology Departments. In the aim of optimizing the adjuvant management and the follow up of the women, working on post-operative specimen is not a problem. However, to provide a preoperative diagnostic tool so as to better stratify women and to optimize their surgical procedures, our analysis should be validated before initial surgery. To do so, two options can be considered: the first one is to assess micro-RNA expression in pre-operative tumor biopsy samples, the second one is to work on pre-operative blood samples. Studying micro-RNA expression profiles on biopsy samples comes with a risk of contamination of normal endometrial or myometrial tissue, which could lead to a wrong analysis. Interestingly, several authors have demonstrated that circulating micro-RNA profiles extracted from plasma samples were correlated with profiles extracted from FFPE specimen [90]. In EC, various studies have demonstrated that circulating micro-RNA could be used as diagnostic tools for endometrioid EC (micro-RNA-15b, -27a, and -233, and micro-RNA-9/micro-RNA-1228 and micro-RNA-9/micro-RNA-92a respectively [91, 92]). Micro-RNA-184, -497, and -196b-3p, have been described in various cancer as promising diagnosis tools preoperatively assessed in blood samples [34, 44, 50, 58, 69, 73, 93, 94]. Owing to their significant implication in EC progression, they may be promising tools of diagnosis, prognosis and treatment response. Micro-RNA 184, who was studied in numerous cancers, appears as a serious candidate for future researches. Although recent technology improvements may allow micro-RNA-184 detection and quantification easily from clinical samples, further investigations are still needed first to assess the exact role of this micro-RNA in endometrial carcinogenesis, second to precise its interactions with the other selected microRNA and third to prove the correlation between blood samples and tumor samples in patients with endometrial cancer.

Some limitations of the study should be underlined. Because of the use of FFPE specimens with insufficient quality and RNA integrity, we could not assess the expression of exact targets for the micro-RNAs validated in this study. Further studies are thus needed to precisely determine the different pathways affected by the selected micro-RNAs. Second, we included patients with both local and distance recurrence that have different long-term prognosis. Indeed, recently, Ouldamer et al. demonstrated that patients recurring under the form of distant metastasis or carcinomatosis had poorer prognosis [95]. Moreover, from clinical point of view this difference is relevant, as patients with local recurrence could have benefited from external beam radiotherapy while patients with distant metastasis or carcinomatosis could have benefited of adjuvant chemotherapy. Third, due to the sample size, it was impossible to determine a combination of micro-RNAs allowing the detection of all R+ patients. Future research is needed to resolve this problem. Finally, the retrospective nature of our data collection, which was focused on the occurrence of recurrence, should be underlined as a possible bias.

## Conclusion

This is, to our knowledge, the first study investigating a correlation between micro-RNA signatures in tumor tissues from LREC women and the occurrence of recurrences.

Our results may provide a basis for further study of the micro-RNA function EC, and be used as a valuable tool for better diagnostic and therapeutic management.

## Abbreviations

EC: endometrial cancer
EMT: epithelial to mesenchymal transition
ESMO: European Society for Medical Oncology
FC: fold change
FFPE: formalin-fixed paraffin-embedded
FIGO: International Federation of Gynecology and Obstetrics
GO: gene ontology
IQR: interquartile range
LREC: low risk endometrial cancer
LVSI: lympho vascular space involvement
qRT–PCR: quantitative real-time PCR
R− women: women whose follow up did not show any recurrence
R+ women: women whose follow up showed recurrences
RFS: recurrence free survival
RT: reverse transcription
